# Case Report: Generalized lipodystrophy following immune-checkpoint inhibitor therapy

**DOI:** 10.3389/fendo.2025.1710985

**Published:** 2025-12-16

**Authors:** Andrew-Hyun Lee, Rina Hui, Jenny E. Gunton

**Affiliations:** 1 Department of Diabetes and Endocrinology, Westmead Hospital, Sydney, NSW, Australia; 2 Faculty of Medicine and Health, The University of Sydney, Sydney, NSW, Australia; 3 Centre of Cancer Medicine, Faculty of Medicine, University of Hong Kong, Hong Kong, Hong Kong SAR, China; 4 Centre for Diabetes, Obesity and Endocrinology Research (CDOER), Westmead Institute for Medical Research, Sydney, NSW, Australia

**Keywords:** lipodystrophy, immunotherapy, checkpoint inhibitor therapy, adipose, diabetes

## Abstract

Immune-related adverse events secondary to immune checkpoint inhibitors (ICI) are increasingly recognised. Lipodystrophy is a rare condition which results in the selective loss of adipose tissue. We describe a case of a 48-year-old woman who had been treated with pembrolizumab for lymph node-positive breast cancer. She was referred to the diabetes service for worsening hyperglycemia, hypertriglyceridemia, and rapid onset of weight loss which occurred a year into pembrolizumab therapy. Examination was consistent with a diagnosis of severe lipodystrophy with severe loss of facial and limb adipose tissue. Investigations including a low leptin level and loss of adiposity on whole body composition analysis were consistent with this diagnosis. A trial of pioglitazone was associated with an improvement in insulin resistance and hypertriglyceridemia, although no improvement in her facial lipodystrophy was observed.

## 1 Introduction

Immune checkpoint inhibitors (ICI) promoting immune-mediated cancer cell death have revolutionized the management of many cancers, and their uses continue to expand. Immune-related adverse events (irAE) are well documented, and occur due to ICIs impairing self-tolerance to native antigens. Endocrinopathies, including thyroid conditions, hypophysitis, adrenal insufficiency, and autoimmune diabetes, are known complications from immune checkpoint inhibitors (ICI) (1–3). Acquired lipodystrophy is a rare complication from ICI therapy which has been documented in fewer than 10 patients worldwide (4–12).

We describe a case of acquired generalized lipodystrophy secondary to pembrolizumab, a monoclonal antibody against programmed death 1 (PD-1), and highlight the possible benefits of thiazolidinedione therapy for management of lipodystrophy and its related complications.

## 2 Case description

A 48-year-old woman was diagnosed with lymph node positive, hormone receptor positive, HER2 (human epidermal growth factor 2) negative, early-stage breast cancer (cT2N1M0). Her medical background included type 2 diabetes mellitus (T2DM) managed on metformin monotherapy and metabolic dysfunction-associated steatotic liver disease (MASLD). She was a non-smoker, and her family history included a sister with breast cancer, and a mother with T2DM.

Her breast cancer was managed with neoadjuvant chemotherapy as part of a clinical trial (with 12 weeks of paclitaxel followed by 4 cycles of doxorubicin and cyclophosphamide every 3 weeks), alongside 8 cycles of neoadjuvant pembrolizumab every 3 weeks from December 2021 to May 2022. Her pathological stage after neoadjuvant treatment was ypT2N0M0. Following surgical resection, she underwent a further 9 cycles of pembrolizumab from September 2022 to February 2023, for another 6 months of immunotherapy. She received in total 1 year of immunotherapy. She also received adjuvant endocrine therapy with goserelin and letrozole in conjunction with her adjuvant pembrolizumab.

While on pembrolizumab therapy, a rapid worsening in her glycemia was noted a year into her cancer therapy, with an increase in HbA1c from 60.7 mmol/mol (7.7%) in April 2022 to 97.8 mmol/mol (11.1%) in December 2022 despite an escalation in her oral hypoglycemic agents, including the addition of sitagliptin and empagliflozin. This coincided with rapid weight loss from 87 kg (body mass index (BMI) 33.2 kg/m^2^) to 66 kg (BMI 25.8 kg/m^2^) over the course of a year (**Table 1**). Additional imaging including CT scans did not identify cancer recurrence as a cause of weight loss.

**Table 1:** Summary of patient’s laboratory test results.

Date	04/07/22	12/07/22	02/09/23	05/04/23	07/05/23	01/31/25	05/20/24	09/02/24	Reference range
Weight (kg)	87	81	75	66	65	65	66	68	–
HbA1c (mmol/mol [%])	60.7 [7.7]	97.8 [11.1]	–	77.0 [9.2]	85.0 [9.9]	66.1 [8.2]	65.0 [8.1]	7.05 [8.6]	20.2-42.1 [4.0-6.0]
Triglycerides (mmol/L)	3.2	–	5.4	–	4.7	5.7	–	2.8	<2.0
LDL-cholesterol (mmol/L)	2.7	–	–	–	–	–	–	2.8	<3.0
HDL-cholesterol (mmol/L)	0.7	–	0.7	–	0.9	0.7	–	0.9	>1.2

The patient was reviewed in the diabetes clinic in August 2023, 20 months after commencement of cancer therapy, at which point her pembrolizumab had been ceased for about 5 to 6 months. Near total loss of adipose tissue in the face and limbs, with some reduced abdominal adipose tissue were found. She had pronounced acanthosis nigricans which on specific questioning she thought had been present for a few months prior to her weight loss.

This clinical presentation was consistent with severe lipodystrophy.

## 3 Diagnostic assessment

**Figure 1:**
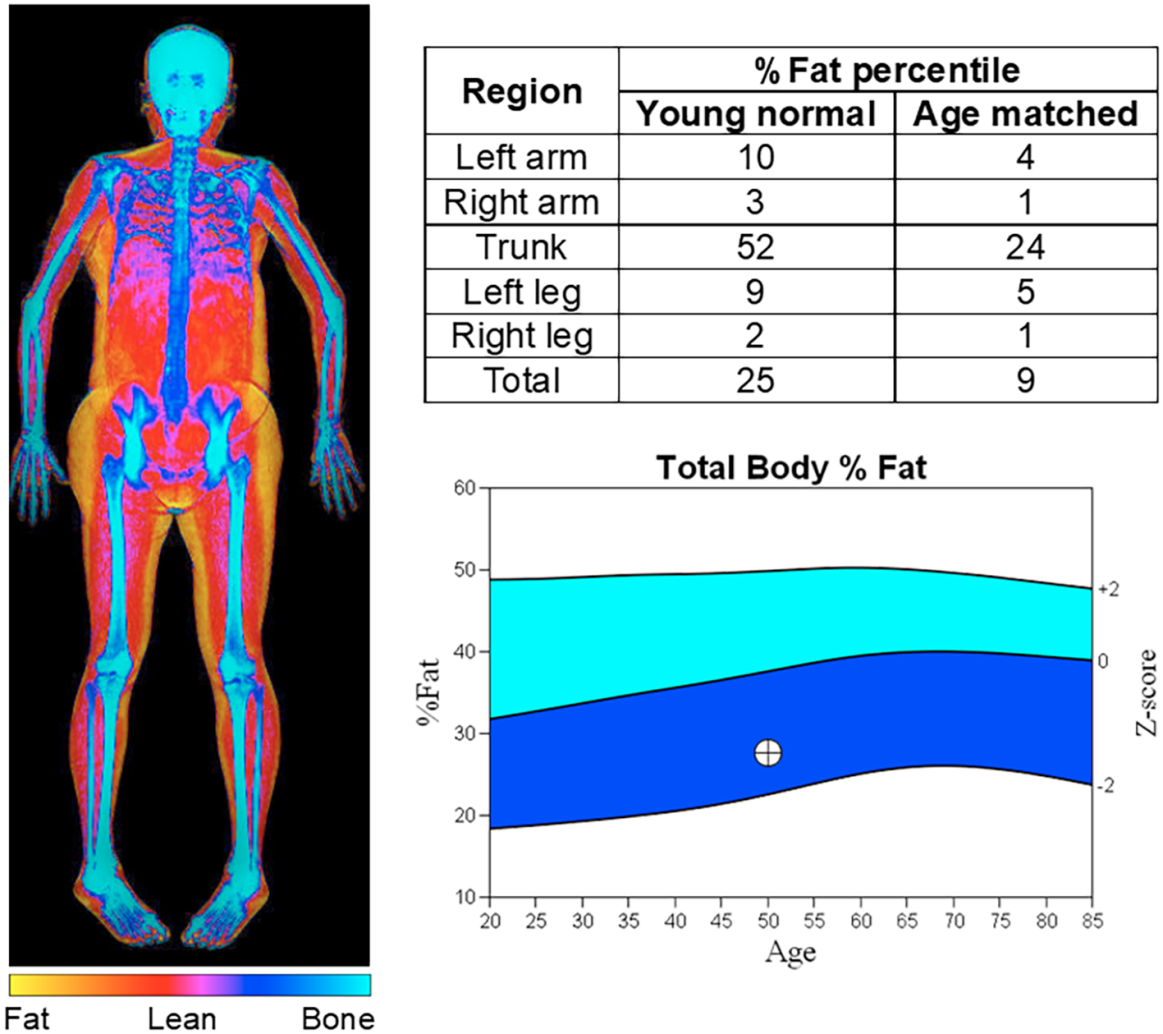
Whole body composition analysis via dual-energy X-ray absorptiometry. False color image representing proportions of adipose, lean mass, and bone tissue (left); numerical fat percentiles by body part (top right); and graphical Z-score of total body fat (bottom right).

**Figure 2:**
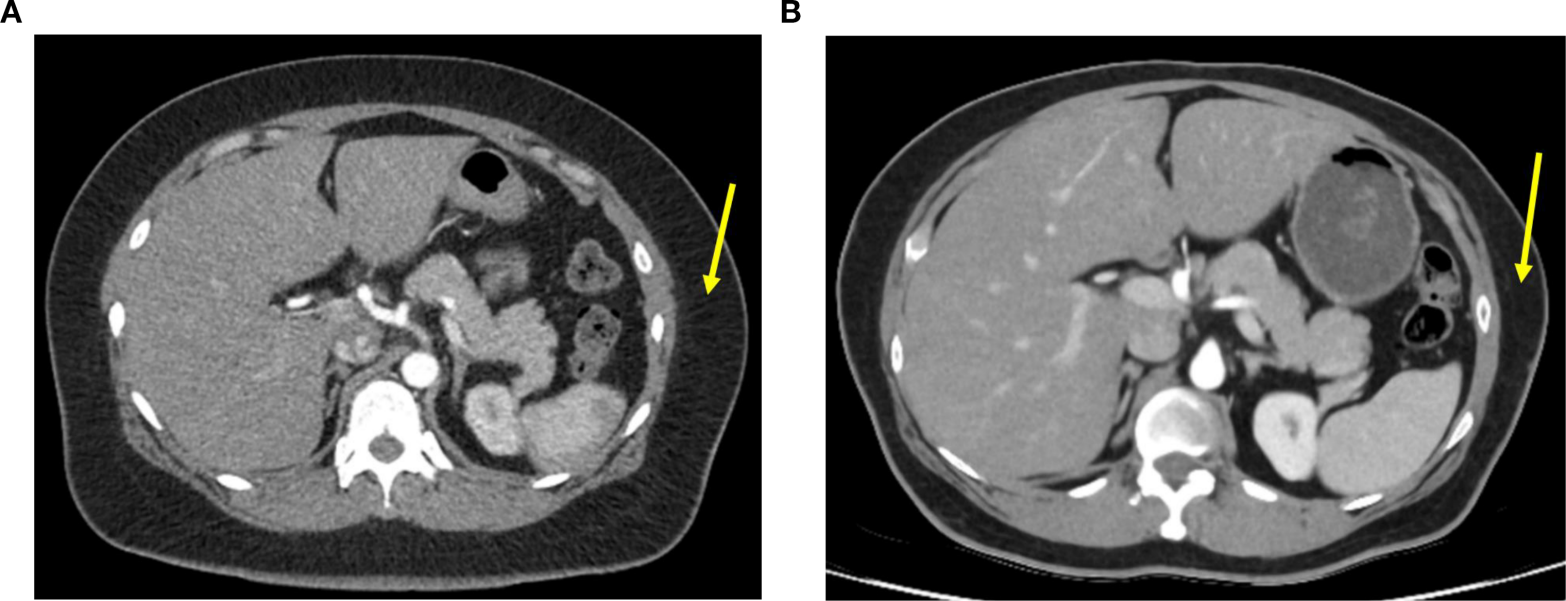
Axial computed tomography slices of the abdomen, demonstrating preserved subcutaneous adipose tissue early after cancer diagnosis in December 2021 (**A**; *arrow = 35 mm of subcutaneous adipose tissue*); and 30 months later in April 2024 demonstrating loss of subcutaneous adipose tissue (**B**; *arrow = 20 mm of subcutaneous adipose tissue*).

Investigations ruled out autoimmune or pancreatogenic diabetes, as well as other endocrine disturbances of the adrenal or thyroid axis. Her leptin level was reduced to 9.92 ng/mL, which was at or below the 10th percentile for age and BMI-matched women respectively (13). Whole body composition dual-energy X-ray absorptiometry revealed loss of adipose tissue to less than the 10th percentile of age-matched controls excluding the trunk (**Figure 1**). A retrospective review of computed tomography images performed for follow up of her malignancy revealed a significant loss of subcutaneous adipose tissue also consistent with lipodystrophy (**Figure 2**).

An associated hypertriglyceridemia had developed, with triglycerides up to 477.9 ng/dL (5.4 mmol/L) (reference range: 177.0 mg/dL [<2.0 mmol/L]). Hepatomegaly was present, with an increase in liver span from 16.4 cm to 17.7 cm on serial imaging over 30 months. Liver elastography revealed marked steatosis of >66% but normal stiffness at 4.8 kPa.

On initial review at the diabetes clinic, the patient’s weight had stabilized at a nadir of 65 kg. She was commenced on gliclazide 120 mg (August 2023) and semaglutide (from September 2023, and increased to 1 mg in January 2024) for management of her diabetes, which improved her HbA1c to 65.0 mmol/mol (8.1%) in May 2024, although further improvements were held back by variable medication adherence.

Pioglitazone 15 mg was then trialed (May 2024), with a marked improvement in the acanthosis nigricans and hypertriglyceridemia 3 months later. Triglycerides improved to 247.8 mg/dL (2.8 mmol/L). A Homeostatic Model Assessment for Insulin Resistance (HOMA-IR) measurement however remained significantly elevated at 23.5 (reference range <2.5) (14), suggestive of ongoing significant insulin resistance. Although she experienced a mild increase in weight to 68 kg, she has yet to demonstrate an increase in weight to her previous baseline. There was no clear change in the appearance of her lipodystrophy.

## 4 Discussion

Lipodystrophy syndromes are rare conditions defined by the selective loss of adipose tissue, which may be partial or generalized depending on the sites of adipose tissue loss. Loss of adipocytes often leads to downstream metabolic complications related to ectopic lipid deposition including insulin resistance, MASLD, hypertriglyceridemia and polycystic ovary syndrome (15).

Acquired generalized lipodystrophy (AGL) is associated with a number of causes, including panniculitis (proposed as type 1 AGL) and autoimmunity (type 2 AGL), while idiopathic AGL is categorized as type 3 (16). Type 2 AGL is associated with other autoimmune conditions including juvenile dermatomyositis, Hashimoto’s thyroiditis, and rheumatoid arthritis (17).

Lipodystrophy secondary to immune checkpoint inhibitors has been described in fewer than ten cases worldwide (4–12), with all but one case presenting with AGL with the exception of one report of partial facial lipodystrophy (10). Truncal involvement has been variable in cases of AGL, with some exhibiting preserved abdominal adiposity similar to our patient (12), with some even describing an increase in abdominal fat (4, 5). Onset of lipodystrophy ranged from 6 weeks to 18 months from commencement of ICIs, with one occurring a few weeks after stopping ICI therapy (10). All cases have been associated with nivolumab or pembrolizumab, and none have occurred with only anti-cytotoxic T-lymphocyte-associated protein 4 therapy. The majority have occurred in the setting of melanoma treatment; our case represents the first in the context of breast cancer.

The pathophysiology of ICI-induced AGL remains unknown, although it is hypothesized to be secondary to autoimmune targeting of adipocytes (type 2 AGL) (9). Alternatively, panniculitis (type 1 AGL) may trigger AGL, with one case presenting with scrotal panniculitis prior to the development of AGL (4), while other cases have noted subclinical panniculitis on histopathology (5, 9, 12).

Diagnosis of lipodystrophy is predominantly clinical, although biochemical and radiological investigations can assist with diagnostic work up. Exclusion of other etiologies can include an autoimmune screen, anti-insulin receptor antibodies, complement levels and myeloma screen (4, 15). Leptin, an adipokine involved in hunger-satiety pathways, is generally reduced, with a greater degree of leptin deficiency associated with complications from lipodystrophy (18). However, absence of hypoleptinemia does not rule out lipodystrophy, with one ICI-induced AGL exhibiting significantly increased leptin levels (9). Another case of ICI-induced AGL demonstrated marked leptin deficiency preceding overt fat loss, suggesting that there may be a possible functional defect in adipocytes prior to adipocyte destruction (6). In our patient, the presence of acanthosis nigricans preceding overt weight loss by over 6 months is in keeping with this hypothesis.

Imaging studies may also be helpful in the work up of lipodystrophy, particularly as patients being treated for cancer often obtain serial imaging. Cross-sectional imaging can help assess the distribution of adipose tissue loss, while functional imaging has been used to support evidence for panniculitis in AGL in other studies, although this was not performed in our patient (6).

Finally, treatment of AGL is primarily directed at managing the metabolic complications of the syndrome. Metformin and insulin are commonly used to manage diabetes, although usually high doses of insulin are required due to the degree of insulin resistance. Thiazolidinediones have garnered interest due to their effect on peroxisome proliferator-activated receptor gamma expressed in adipose tissue, with these agents shown to promote adipogenesis and lipid storage (18, 19). An improvement in insulin resistance, hypertriglyceridemia, hepatic volume and steatosis in partial lipodystrophy has been demonstrated with thiazolidinediones (20), although their efficacy has been less well studied in generalized cases. As the principal site of action of thiazolidinediones is adipose tissue, efficacy of these agents can be expected to be significantly reduced in cases of generalized lipodystrophy due to profound loss of adipose. In our case, the presence of residual adipose tissue, particularly in the abdominal region, may have increased her response to pioglitazone.

In ICI-related cases, only two out of four cases which reported using pioglitazone commented on its effect, with one suggesting a possible improvement in triglycerides and liver fat content (11), whereas the other did not note any benefit after several weeks (6). In our patient, pioglitazone was the only agent to cause a significant improvement in her acanthosis nigricans and hypertriglyceridemia, although no clear benefit to weight gain or cosmesis was attained.

Metreleptin, a leptin analogue, is approved in various countries for leptin deficiency in non-HIV-related lipodystrophy and has been shown to improve metabolic complications in AGL (21). Its use however remains limited, particularly in the setting of ICI and cancer therapy, with possible associations of leptin therapy with immune effects on T cells, as well as unclear relationships with T cell lymphoma and activation of autoimmune renal or hepatic disease (22).

## 5 Conclusion

In summary, acquired generalized lipodystrophy is an increasingly recognized but rare complication from immune checkpoint inhibitor therapy. Significant weight loss with the onset of new or worsening metabolic disturbances in the absence of cancer recurrence should prompt targeted examination and diagnosis of this condition. Adipogenesis that may occur with thiazolidinediones may be effective in targeting the insulin resistance and hypertriglyceridemia that occur as a result of lipodystrophy. We speculate that early thiazolidinedione use could possibly reduce the cosmetic and metabolic consequence.

## Data Availability

The raw data supporting the conclusions of this article will be made available by the authors, without undue reservation.
